# 3D-printed porous scaffold promotes osteogenic differentiation of hADMSCs

**DOI:** 10.1515/med-2021-0233

**Published:** 2021-08-24

**Authors:** Xuebin He, Huafei Ao, Ying Qiao, Zhengwen Li

**Affiliations:** Ear-Nose-Throat Department, Shanghai Pudong Hospital, Fudan University Pudong Medical Center, Shanghai 201399, China

**Keywords:** 3D-printed porous titanium alloy scaffold, hsa_circ_0019142, hADMSCs, osteogenic differentiation

## Abstract

**Objective:**

To explore the role of a three-dimensional (3D)-printed porous titanium alloy scaffold (3D scaffold) in the osteogenic differentiation of human adipose-derived mesenchymal stem cells (hADMSCs) and the underlying mechanism.

**Methods:**

hADMSCs were divided into control and 3D scaffold groups. The osteogenic differentiation of hADMSCs and expression of osteogenic makers were estimated. Based on the information from published articles, five candidate circular RNAs were selected, and among them, hsa_circ_0019142 showed the most promising results. Finally, control group cells were overexpressed or silenced with the hsa_circ_0019142. Then, Alizarin red S (ARS) staining, calcium content analysis and estimation of alkaline phosphatase (ALP), osteocalcin (OCN), runt-related transcription factor 2 (RUNX2), and collagen-1 (COL1) were performed to evaluate the role of hsa_circ_0019142 on osteogenic differentiation.

**Results:**

Osteogenic differentiation of the hADMSCs was significantly higher in the 3D scaffold group than in the control group, as evidenced by ARS staining, increased calcium concentration, and elevated expression of above four osteogenic factors. qPCR revealed that the expression of hsa_circ_0019142 was significantly higher in the 3D scaffold group. Overexpression of hsa_circ_0019142 promoted the osteogenic differentiation of hADMSCs, while knockdown of hsa_circ_0019142 caused the opposite results.

**Conclusion:**

The 3D-printed scaffold promoted osteogenic differentiation of hADMSCs by upregulating hsa_circ_0019142.

## 1 Introduction

Replacement and regeneration of bone defects that occur following trauma, osteoarthritis, tumors, metabolic disorders, and osteoporosis poses significant challenges [1]. Bone grafting (autologous and allografting) plays an important role in the field of orthopedics; however, the chance of disease transmission (in allografting) and donor-related issues, such as impaired mobility and unavailability of a suitable donor, limit the widespread application of grafting [2,3]. Regenerative medicine aims to address this issue, specifically through the integration of biological, medical, and engineering principles [4]. The focus is on the maintenance and improvement in specific organ functions through the replacement and/or repair of specific organs [4,5]. The conventional two-dimensional (2D) cell culture does not allow interaction between the cells and extracellular matrix [6]. Thus, three-dimensional (3D) scaffolds composed of stem cells and other biomolecules are gaining popularity [7].

Bone tissue engineering, using titanium and its alloys such as Ti6Al4V, has been widely used in the fields of dentistry and orthopedics [8]. However, the elastic modulus of dense titanium is far greater than that of natural bones (Ti: 110 GPa and Ti6Al4V: 125 GPa vs cancellous bone: 1.5–11.2 GPa and cortical bone: 7–20 GPa), leading to increased bone resorption and implant failure [9]. Hence, compared to dense titanium, porous titanium appears to be far more promising owing to its mechanical properties; the porous nature of the scaffold favors circulation of body fluids, including blood, and transportation of nutrients and oxygen [3]. Besides these benefits, the pores also allow bone regeneration by providing space for the same, better bonding between the tissue and metal, and more efficient transfer of the load, thus avoiding stress shielding effects and prolonging the life of the implant in the long term [3,8,9]. Recently published reports indicated the potential of 3D scaffolds in many medical conditions including wound healing, cardiovascular diseases, and bone regeneration [10,11,12].

Circular RNAs (circRNAs) are abundant in the cells and are evolutionally conserved [13]. There is a growing consensus that circRNAs are not only the by-products of splicing, but also a new class of non-coding RNAs (ncRNAs). NcRNAs, including the housekeeping RNAs such as rRNA, tRNA, and small nucleolar RNA, are considered as a class of RNAs that do not encode proteins. Many of the circRNAs act as microRNA (miRNA) sponges, and thus, play an important role in gene expression [14]. Current evidence revealed circRNAs as potential biomarkers with high prognostic and diagnostic values for different diseases, such as cardiovascular diseases, neurological disorders, and malignancies [15]. Additionally, circRNAs involved in bone regeneration were recently found to be also involved in the regulation of osteogenic differentiation [16,17].

3D printing technology is a burgeoning industry that aims to provide personalized and precise solutions according to the requirements of clinical patients and experimental studies. In this study, a 3D porous titanium alloy scaffold (3D scaffold) was manufactured by 3D printing and implanted with human adipose-derived mesenchymal stem cells (hADMSCs) to evaluate the effects of the 3D scaffold on osteogenic differentiation of the hADMSCs, and the circRNAs responsible for this process were also investigated.

## 2 Materials and methods

### 2.1 3D printing and scaffold preparation

First, Ti6Al4V films (diameter of 14 mm and thickness of 1 mm) were ultrasonically cleaned in acetone, ethanol, and deionized water (dH_2_O) separately before use. Next the scaffold was prepared using a selective laser melting 3D printing machine (EOSINT M280, EOS Ltd., Munich, Germany). Before incubating the cells, the scaffolds were ultrasonically cleaned with acetone, ethanol, and dH_2_O separately and finally dried using gaseous nitrogen.

### 2.2 Cell culture

The hADMSCs were purchased from the Cell Bank of the Chinese Academy of Sciences. The cells were cultured in alpha-MEM medium (Life Technologies, USA), supplemented with 10% of fetal bovine serum, and maintained in a humidified chamber in 5% of CO_2_ at 37°C. The medium was replaced with fresh medium every three days. The cells were separated into two groups, the control and test (3D scaffold) groups. Cells of the control group were seeded in the 12-well plate directly, while cells of the test group were seeded in the 3D scaffold that was placed in the 12-well plate. The cells of both groups were cultured in osteogenesis induction medium, supplemented with 100 nM of dexamethasone (Sigma-Aldrich, Saint Louis, MO), 0.2 mM of ascorbic acid (Sigma-Aldrich), 10 mM of β-glycerophosphate (Sigma-Aldrich), and 50 ng/mL of bone morphogenetic protein-2 (BMP-2, R&D Systems, MN, USA), for 14 days.

### 2.3 Cell collection

To compare the effects of the 3D scaffold on osteogenic differentiation of the cells from the two different groups (the control and 3D scaffold groups), the cells in the plate and 3D scaffold were digested with trypsin. The cell density and living cell rate were determined using Countstar^®^ BioTech (Countstar, Shanghai, China). Then, equivalent living cells collected from the two groups were used for further evaluation.

### 2.4 Mineralization assay

The collected cells were seeded in 12-well culture plates overnight, fixed with 4% of paraformaldehyde for 30 min and then incubated in a solution of 0.1% Alizarin red S (ARS; Sigma-Aldrich) at pH of 4.2 in the dark for 20 min to stain the calcified nodules. To evaluate the effects of hsa_circ_0019142 on calcium deposition, the cells were seeded in 12-well culture plates and cultured in osteogenic medium for 14 days. Then, the cells were fixed and stained, as described previously. Calcium concentration of each sample was determined using a calcium assay kit (MAK022, Sigma-Aldrich), following the manufacturers’ instructions.

### 2.5 Transfection assay

The hADMSCs were transfected with either the overexpression vector of hsa_circ_0019142 or with the negative control vector (NC; GenePharma, Shanghai, China) using Lipofectamine^®^ 2000 Transfection Reagent (Life Technologies, USA), according to the manufacturer’s instructions. For knockdown of hsa_circ_0019142 in hADMSCs, cells were transfected with si-hsa_circ_0019142 (5′-ACA AAC GGT TGA ACT GGC AAT-3′) and si-NC (5′-UUC UCC GAA CGU GUC ACG UTT-3′), using Lipofectamine^®^ 2000 Transfection Reagent following the manufacturer’s instructions.

### 2.6 Quantitative polymerase chain reaction (qPCR)

RNA was isolated using the TRIzol RNA reagent (Invitrogen, Carlsbad, CA), according to the manufacturer’s instructions. The RNase-Free DNase Set (Qiagen) was used to eliminate any contamination of the genomic DNA. For the synthesis of complementary DNA , total RNA (1 µg) was reverse transcribed using random primers and the reverse transcriptase enzyme (Applied Biosystems, Foster City, CA). Subsequently, qPCR was performed using the Power SYBR Green PCR Mastermix (Applied Biosystems, Foster City, CA, USA) on an Applied Biosystems 7500 Fast Real-time PCR System. The reaction was conducted in triplicate for each gene. The human gene-specific primers are listed in Table 1.

**Table 1:** The primers used for qPCR

circRNAs/genes	Forward (5′ → 3′)	Reverse (5′ → 3′)
hsa_circ_0131461	CAAGAGGAATCCAGCAAAGC	CATGGAGAGAGGACCTGAGC
hsa_circ_0026827	CTCGGCACTTAGCATCATCA	TCCATGTTTCTGCTCCTGTG
hsa_circ_0011173	ACCTCTTAAAGGGCGAGAGC	GAGGTTCTTGTTGGCCTGAG
hsa_circ_0019142	TTTCCAGGAAGCAGCAGGAT	ATACGCTGGACTCAGGTTGG
hsa_circ_0019236	ATATAAAATCCGGCCCCTCA	CTTCAGCTTCCTCTGCTTCC
ALP	GTGAACCGCAACTGGTACTC	GAGCTGCGTAGCGATGTCC
OCN	CACTCCTCGCCCTATTGGC	CCCTCCTGCTTGGACACAAAG
RUNX2	TGGTTACTGTCATGGCGGGTA	TCTCAGATCGTTGAACCTTGCTA
COL1	GAGGGCCAAGACGAAGACATC	CAGATCACGTCATCGCACAAC
β-Actin	CATGTACGTTGCTATCCAGGC	CTCCTTAATGTCACGCACGAT

### 2.7 Western blot

Cell lysates were prepared using the cell lysis buffer (Sigma-Aldrich) and centrifuged (13,800*×g* for 5 min at 4°C). Total protein concentration was measured using the Bradford protein assay kit from Hangzhou MultiSciences (Lianke) Biotech Co., Ltd. (Hangzhou, China), according to the manufacturer’s instructions. Protein (20 μg) was separated on 12% SDS-PAGE gels and then transferred to polyvinylidene fluoride membranes. The membranes were incubated with the respective monoclonal antibodies at 4°C overnight and then incubated with the secondary antibody at room temperature for 1 h; β-actin was used as a loading control.

### 2.8 Statistical analysis

Data are presented as mean values ± standard deviation. One-way ANOVA and Student–Newman–Keuls post hoc test were performed for data analysis. The SPSS 17.0 (Chicago, IL, USA) was used to analyze the data. *P* < 0.05 was considered statistically significant.

## 3 Results

### 3.1 3D-printed titanium alloy scaffold promoted osteogenic differentiation of the hADMSCs

**Figure 1:**
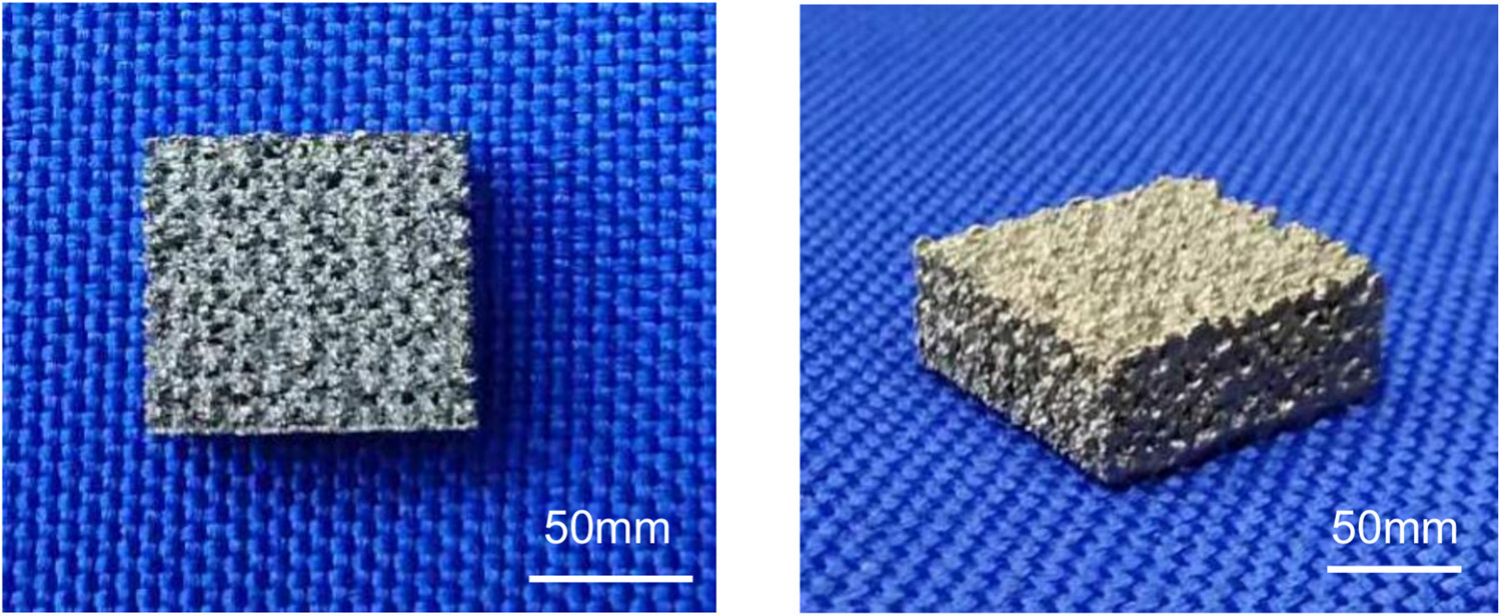
The 3D-printed porous titanium alloy scaffold (parameters: 100 mm × 90 mm × 45 mm). 3D, three-dimensional.

**Figure 2:**
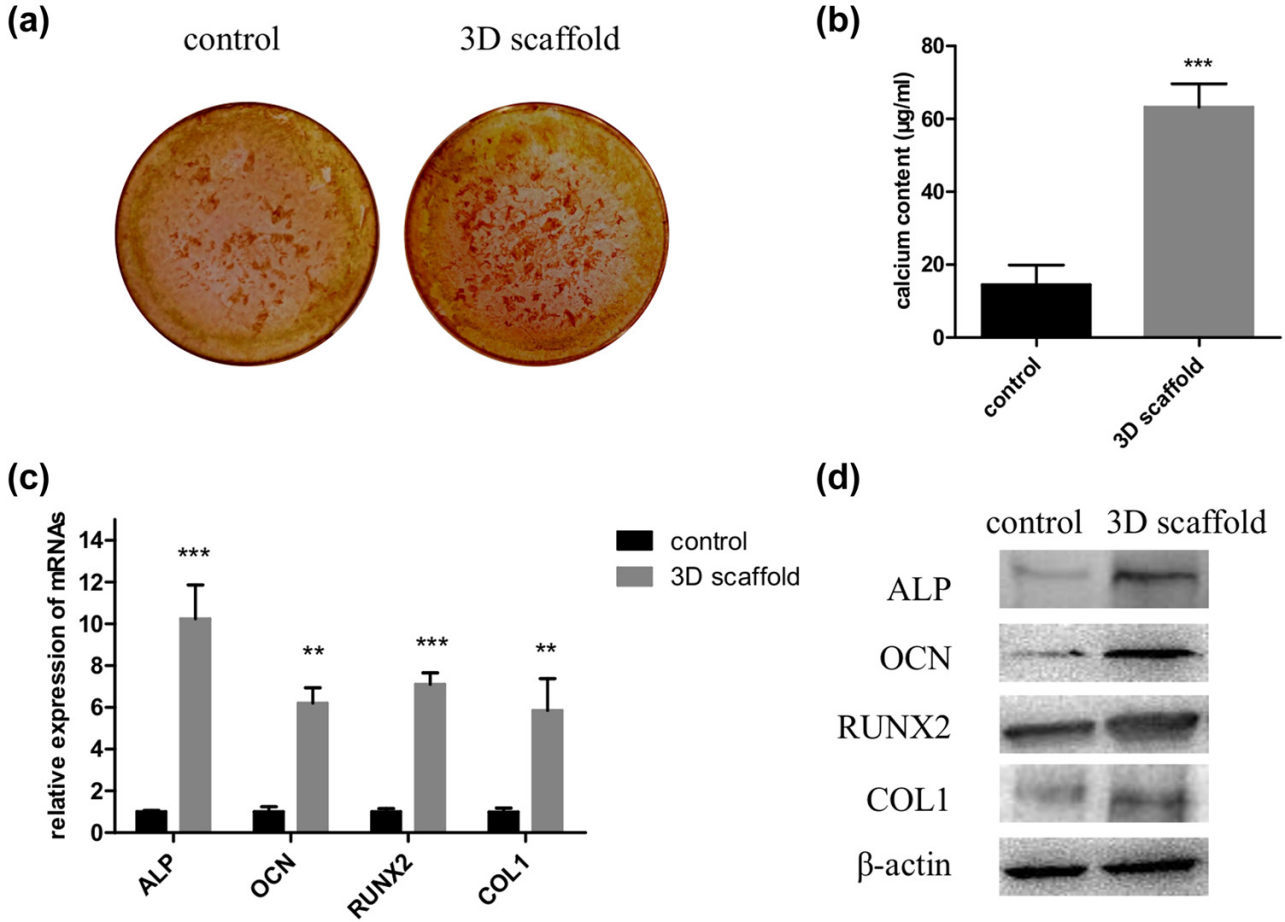
The 3D scaffold promotes osteogenic differentiation of hADMSCs. Cells were seeded in a 2D culture plate (control) and the 3D scaffold (test) to induce osteogenesis; then, they were digested and collected for ARS staining (a), calcium concentration analyses (b), and detection of relative mRNA (c) and protein levels of ALP, OCN, RUNX2, and COL1 (d) (*n* = 3 in each experiment). 3D, three-dimensional; 2D, two-dimensional; ARS, Alizarin red S. **P < 0.01 and ***P < 0.001.

A 3D scaffold with a dense porous structure was successfully printed (Figure 1a). The 3D scaffold was seeded with cells and then cultured in the induction medium for osteogenic differentiation. ARS is an anthraquinone dye, which has been widely used to assess calcium-rich deposits in cell cultures. Both the control and 3D scaffold groups were stained with ARS (Figure 2a). Next we measured the calcium concentration in the cells; it was significantly higher (*P* < 0.001) in the 3D scaffold group than in the control group (Figure 2b).

### 3.2 Estimation of the osteogenic activity of the 3D scaffold

To assess the osteogenic activity of the two cell groups, we measured the mRNAs of the osteogenesis markers, including runt-related transcription factor 2 (RUNX2), collagen protein 1 (COL1), osteocalcin (OCN), and alkaline phosphatase (ALP), using qPCR. Expression of the mRNAs was significantly increased in the 3D scaffold group compared to the control group (*P* < 0.001 for ALP and RUNX2 and *P* < 0.01 for OCN and COL1) (Figure 2c). Next western blot analysis demonstrated that the expression of the corresponding proteins were visibly higher in the 3D scaffold group than in the control group (Figure 2d).

### 3.3 Expression levels of the circRNAs in the hADMSCs that were cultured in the 3D scaffold

**Figure 3:**
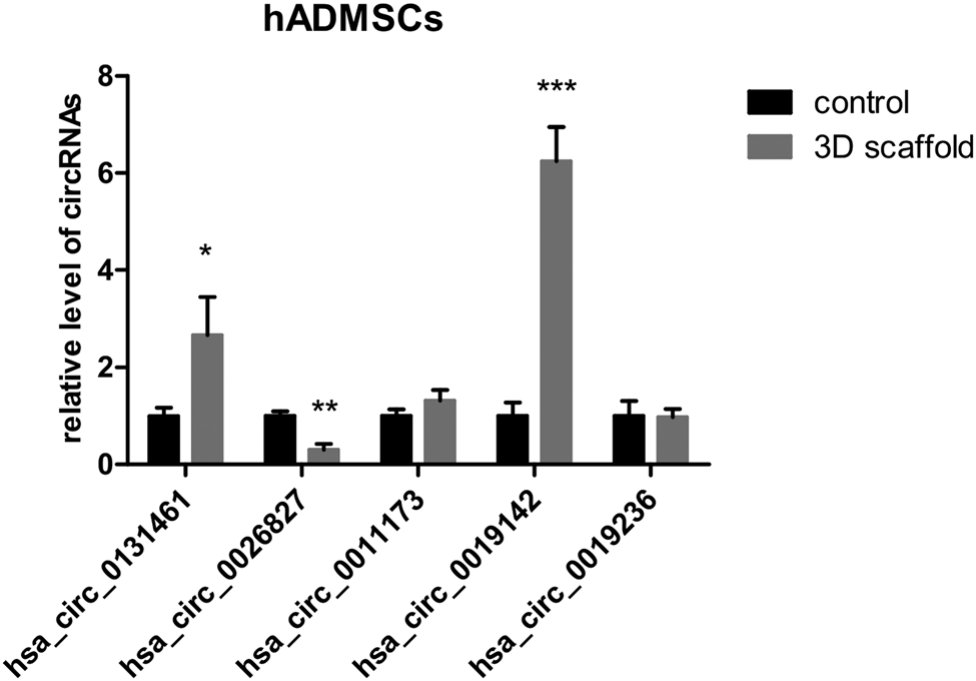
Relative expression of circRNAs in the 3D scaffold group. Cells were seeded in a 2D culture plate (control) and the 3D scaffold (test) to induce osteogenesis; then, they were digested and collected to analyze the relative levels of the five candidate circRNAs (*n* = 3). 3D, three dimensional; 2D, two dimensional. *P < 0.05, **P < 0.01, and ***P < 0.001.

qPCR revealed that among the five candidate circRNAs, hsa_circ_0131461 and hsa_circ_0019142 showed significantly increased expression levels and hsa_circ_0026827 showed a significantly reduced expression level in the 3D scaffold group (*P* < 0.05, *P* < 0.001, and *P* < 0.01, respectively) (Figure 3). Since the fold change (FC) of hsa_circ_0019142, among the FCs of the three circRNAs, was the highest (*P* < 0.001), hsa_circ_0019142 was chosen for further study.

### 3.4 Overexpression of hsa_circ_0019142 promoted osteogenic differentiation of the hADMSCs

**Figure 4:**
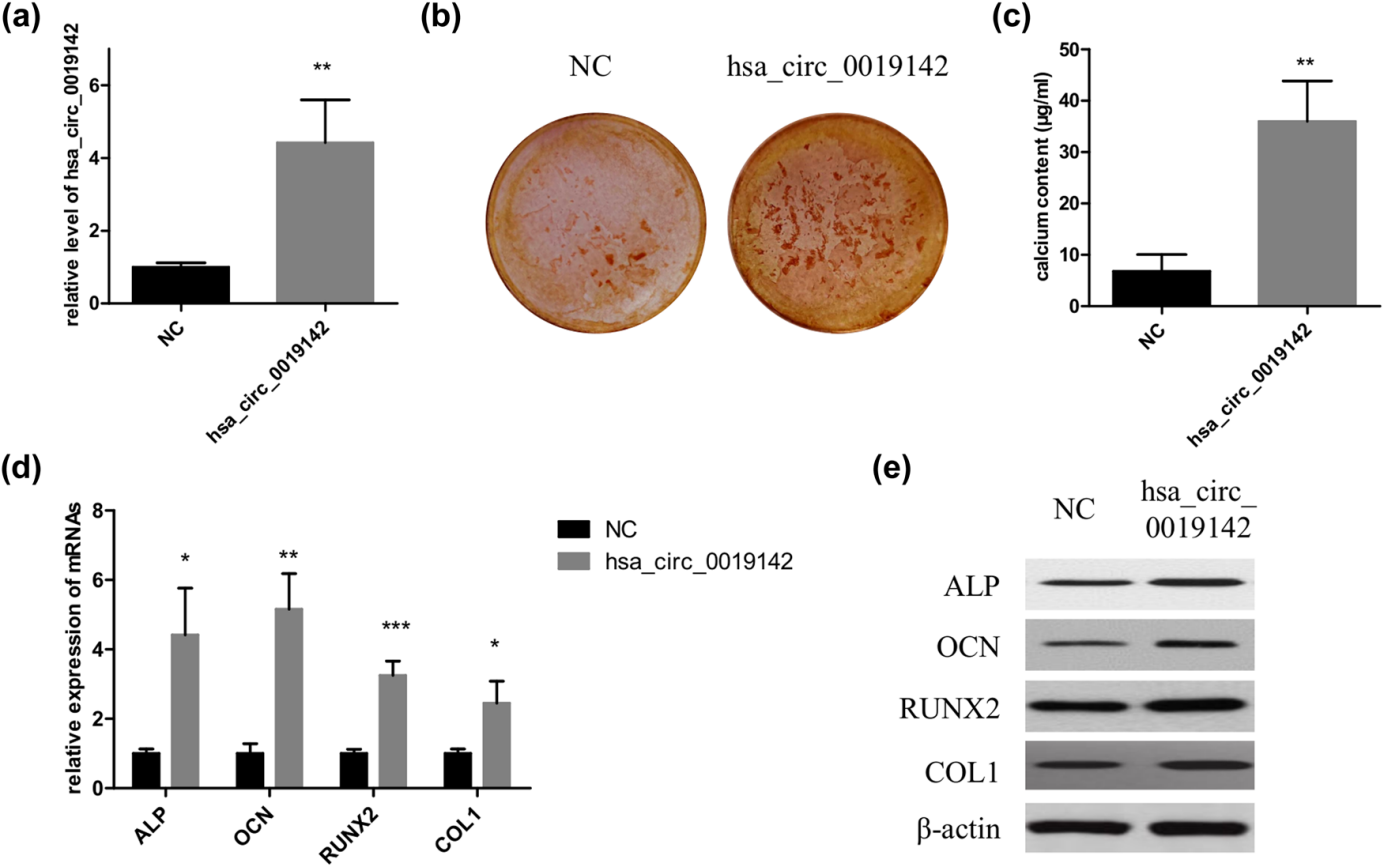
Overexpression of hsa_circ_0019142 promotes osteogenic differentiation of hADMSCs. (a) qPCR verification of the level of hsa_circ_0019142 in cells overexpressing it. After the induction of osteogenesis in the hsa_circ_0019142 overexpressed cells in the culture plate, the cells were used for ARS staining (b), calcium concentration analyses (c), and detection of relative mRNA (d) and protein levels of ALP, OCN, RUNX2, and COL1 (e) (*n* = 3 in each experiment). NC, negative control; ARS, Alizarin red S. *P < 0.05, **P < 0.01, and ***P < 0.001.

After the hADMSCs were transfected with the overexpression vector of hsa_circ_0019142, successful transfection was confirmed by qPCR. Expression of hsa_circ_0019142 was found to be significantly higher (*P* < 0.01) in the cells transfected with hsa_circ_0019142 than in those transfected with the NC vector (Figure 4a). Next the cells were seeded in 12-well culture plates to induce osteogenesis. ARS staining revealed greater osteogenic activity in the hsa_circ_0019142 overexpressed cells than that in the NC cells (Figure 4b). Similarly, the calcium concentration was found to be significantly higher (*P* < 0.01) in the hsa_circ_0019142 overexpressed cells than in the NC cells (Figure 4c). The expression of the different osteogenesis markers (ALP, OCN, RUNX2, and COL1) was significantly elevated in the hsa_circ_0019142 overexpressed cells than in the NC cells (*P* < 0.05 for ALP and COL1, *P* < 0.01 for OCN, and *P* < 0.001 for RUNX2) (Figure 4d). This was further confirmed by the similar results obtained from the western blot analysis (Figure 4e).

### 3.5 Knockdown of hsa_circ_0019142 inhibited osteogenic differentiation of the hADMSCs

**Figure 5:**
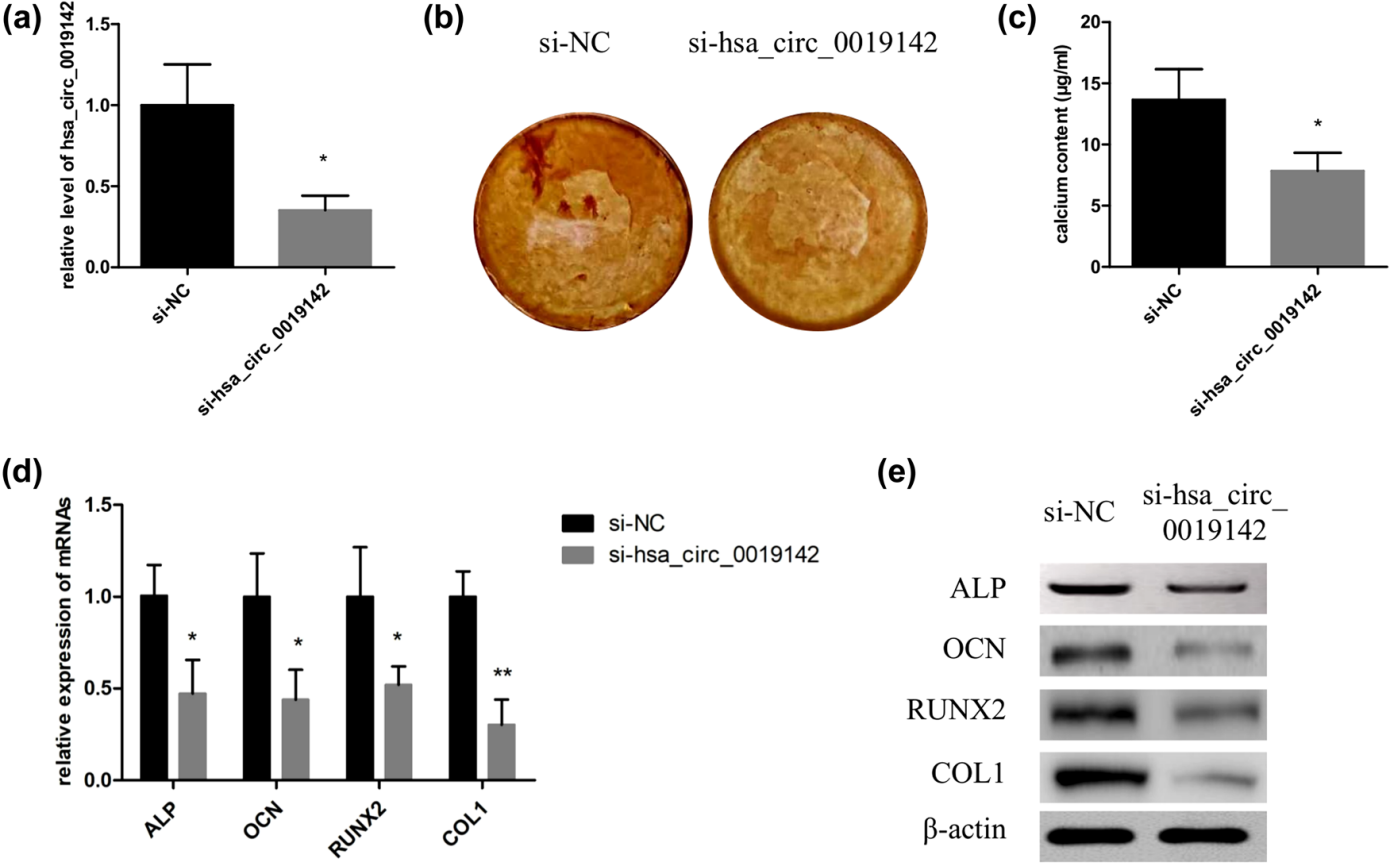
Knockdown of hsa_circ_0019142 inhibits osteogenic differentiation of hADMSCs. (a) qPCR verification of the level of hsa_circ_0019142 in cells silencing it. After the induction of osteogenesis in the hsa_circ_0019142 silenced cells in the culture plate, the cells were used for ARS staining (b), calcium concentration analyses (c), and detection of relative mRNA (d) and protein levels of ALP, OCN, RUNX2, and COL1 (e) (*n* = 3 in each experiment). NC, negative control; ARS, Alizarin red S. *P < 0.05 and **P < 0.01.

qPCR result in Figure 5a indicated that the expression level of hsa_circ_0019142 was successfully silenced in hADMSCs. After the induction of osteogenic differentiation, ARS staining results indicated that the mineralization in si-hsa_circ_0019142 group was significantly lower (*P* < 0.01) than that in si-NC group (Figure 5b and c). Besides, the mRNA expression levels of ALP, OCN, RUNX2, and COL1 were all obviously reduced after the hsa_circ_0019142 was silenced in hADMSCs (Figure 5d), and the protein levels of the four markers were also decreased (Figure 5e).

## 4 Discussion

A vast majority of the world’s population suffers from diseases that can be successfully managed, and even cured in some cases, through the transplantation of organs or tissues, but the lack of availability of tissues/organs remains one of the greatest challenges [18]. Hence, searching for an alternative to bone grafting has taken a prominent seat in the field of regenerative medicine. In the present study, osteogenic differentiation of the hADMSCs in the 3D scaffold and the underlying mechanism were studied for the first time, aiming to provide a potential solution for bone regeneration.

Besides being multipotent, the hADMSCs have self-renewal properties; they can differentiate into adipocytes [19], osteoblasts [19], and chondrocytes [20]. The hADMSCs are advantageous compared to other stem cells because they are easily accessible and can be obtained in large amounts through subcutaneous liposuction [21] without ethical or political issues as these mesenchymal stem cells are obtained from autologous fat [22]. Therefore, hADMSCs were used in the present study. Stem cell differentiation is an important strategy in regenerative medicine. BMPs are involved in multiple processes, including the regulation of osteogenesis. BMP-2 is known to enhance the osteogenic differentiation of mesenchymal stem cells obtained from various locations; hence it was supplied in the osteogenic differentiation medium to assist in osteogenesis.

Our results indicated that osteogenic mineralization of the hADMSCs was successfully induced after treatment of both the 2D plate and 3D scaffold with BMP-2. Moreover, hADMSCs in the 3D scaffold group possessed stronger osteogenic differentiation potentials and showed increased calcium concentration and higher expression of the osteogenesis-related factors, such as RUNX2, COL1, OCN, and ALP, than that of the cells in the control group. This indicated a significant advantage of the 3D scaffold in osteogenic induction, and this could be attributed to the unique titanium alloy material or porous structure of the scaffold [23]. Notably, it was difficult to compare the results of ARS staining of the two groups directly. Hence, the cells were collected after osteogenic induction, which are also required for further analyses of mRNA and protein levels of the markers. Additionally, the cell number and living cell rate were calculated after the cells were collected from the two groups. Only equivalent living cells were used for further evaluation, ensuring the reasonability in the comparisons and good repeatability in the study.

Recently, a review on the prospects of circRNAs in osteogenesis demonstrated that different circRNAs were involved in the modulation (upregulation or downregulation) of different signaling pathways and proteins that were associated with osteogenesis [24], highlighting the important role of circRNAs in osteogenesis. The relationship between the circRNAs and osteoblastic differentiation of stem cells has also been evidenced in previous studies. Zhang et al. identified 3938 upregulated and 1505 downregulated circRNAs after the osteoblastic differentiation of human bone marrow stem cells [25]. Peng et al. explored the role of hsa_circRNA_33287 in the osteogenic differentiation of maxillary sinus membrane stem cells [26]. The overexpression and silencing of hsa_circRNA_33287 increased and decreased the expression levels of the key osteogenesis markers (including RUNX2, osterix, and ALP), respectively. Hence, circRNAs might also play a role in the osteogenic differentiation prompted by the 3D scaffold. To obtain the candidate circRNAs, we focused on two important circRNA expression profiles, which were identified from the osteogenic differentiation of bone marrow stem cells [25] and MC3T3-E1 cells [27], presenting plenty of potential circRNAs that were involved in osteogenic differentiation. Five circRNAs, among the top eight differentially expressed circRNAs in each profile, were then randomly selected as candidate circRNAs.

qPCR results indicated that three of the five candidates significantly changed in the 3D scaffold group, and hsa_circ_0019142, which showed the highest FC, was chosen for further confirmation studies. The results revealed that calcium concentration and expression levels of RUNX2, COL1, OCN, and ALP were significantly increased in the hsa_circ_0019142 group indicating an enhanced osteogenic differentiation of the hADMSCs due to the overexpression of hsa_circ_0019142, while knockdown of hsa_circ_0019142 led to the opposite results. Peng et al. also showed that hsa_circRNA_33287 acted as a molecular sponge for miR-214-3p, regulating RUNX3 expression by targeting its 3′-UTR region and thus promoting ectopic bone formation [26]. MiRNA sponging is one of the important mechanisms by which circRNA exerts its functions [13,14,15]. Hence, the promoted effect of hsa_circ_0019142 on the osteogenic differentiation of the hADMSCs might also be dependent on the direct regulation of the objective miRNA and target genes by circRNA. However, this finding still requires further study.

In conclusion, the present study proved that hADMSCs, which were seeded in the 3D-printed porous titanium alloy scaffold, possessed higher osteogenic differentiation potentials due to the upregulation of hsa_circ_0019142.
